# The impact of closed incision negative pressure therapy on prevention of median sternotomy infection for high risk cases: a single centre retrospective study

**DOI:** 10.1186/s13019-020-01265-1

**Published:** 2020-08-19

**Authors:** Rona Lee Suelo-Calanao, Richard Thomson, Maxine Read, Euan Matheson, Emmanuel Isaac, Mubarak Chaudhry, Mahmoud Loubani

**Affiliations:** 1 Department of Cardiothoracic Surgery, Castle Hill Hospital, Cottingham, HU16 5JQ UK; 2 Royal Infirmary of Edinburgh, Edinburgh, UK

**Keywords:** Sternal wound infection, Closed incision negative pressure therapy, Sternotomy

## Abstract

**Background:**

Sternal wound infection (SWI) following cardiothoracic surgery is a major complication. It may significantly impact patient recovery, treatment cost and mortality rates. No universal guideline exists on SWI management, and more recently the focus has become prevention over treatment. Recent studies report positive outcomes with closed incision negative pressure therapy (ciNPT) on surgical incisions, particularly for patients at risk of poor wound healing.

**Objective:**

This study aims to assess the effect of ciNPT on SWI incidence in high-risk patients.

**Methods:**

A retrospective study was performed to investigate the benefit of ciNPT post sternotomy. Patients 3 years before the introduction of ciNPT (Control group) and 3 years after ciNPT availability (ciNPT group) were included. Only patients that had two or more of the risk factors; obesity, Chronic Obstructive Pulmonary Disease, old age and diabetes mellitus in the High Risk ciNPT cohort were given the ciNPT dressing. Patient demographics, EuroSCOREs and length of staywere reported as mean ± standard deviation. The Fisher’s exact test (two-tailed) and an unpaired t-test (two-tailed) were used to calculate the *p*-value for categorical data and continuous data, respectively.

**Results:**

The total number of patients was 1859 with 927 in the Control group and 932 in the ciNPT group. No statistical differences were noted between the groups apart from the Logistic EuroSCORE (Control = 6.802 ± 9.7 vs. ciNPT = 8.126 ± 11.3; *P* = 0.0002). The overall SWI incidence decreased from 8.7 to 4.4% in the overall groups with the introduction of ciNPT (*P* = 0.0005) demonstrating a 50% reduction. The patients with two and above risk factor in the Control Group (High Risk Control Group) were 162 while there was 158 in the ciNPT Group (High Risk ciNPT Group). The two groups were similar in all characteristics. Although the superficial and deep sternal would infections were higher in the High Risk Control Group versus the High Risk ciNPT group patients (20(12.4%) vs 9(5.6%); *P* = 0.049 respectively), the length of postoperative stay was similar in both (13.0 ± 15.1 versus 12.2 ± 15.6 days; p + 0.65). However the patients that developed infections in the two High Risk Groups stayed significantly longer than those who did not (25.5 ± 27.7 versus 12.2 ± 15.6 days;*P* = 0.008). There were 13 deaths in Hospital in the High Risk Control Group versus 10 in the High Risk ciNPT Group (*P* = 0.66).

**Conclusion:**

In this study, ciNPT reduced SWI incidence post sternotomy in patients at risk for developing SWI. This however did not translate into shorter hospital stay or mortality.

## Background

Sternal wound infection (SWI) is a devastating postoperative complication for any patient [1]. Despite the recent advances in cardiac surgery, the rate of sternal wound complications has seen minimal improvement [2]. SWI has a reported incidence between 0.5 and 10% and has an associated 1-year mortality of 0.5 to 9% [2, 3]. We have reported previously a deep sternal wound infection (DSWI) incidence of 0.59% with an associated 1-year mortality rate of 9.1% with older age, obesity, diabetes and chronic obstructive pulmonary disease (COPD) as the associated risk factors in our department [4].

There is currently no universal guideline for the treatment of SWI [1]. However, recent studies have reported a reduced incidence of SWI complications in centres utilizing closed incision negative pressure therapy (ciNPT) [2]. The ciNPT system is a closed system that can maintain an intact environment. Compared to a standard sterile dressing, the ciNPT canister can hold excess fluids removed from the incision. It may be used over anatomically challenging incision locations whilst maintaining its seal, thus minimizing the risk from external contamination. The ciNPT unit is small so a patient can mobilise freely and the ciNPT dressing is waterproof allowing the patient to shower with the dressing in place [5].

This study aims to assess the effect of ciNPT use over a closed incision post-median sternotomy on the incidence of sternal wound infection (SWI) in high-risk patients with two or more risk factors with focus on the hypothesis that the use of ciNPT would reduce the rate of infection.

## Materials and methods

### Study design

A retrospective study was performed to evaluate the clinical benefit of ciNPT. Patient cohort data were acquired from the Department of Cardiothoracic Surgery and were collected into our patient analysis and tracking database. The study received approval from the Institutional Review Board.

### Study patients/patient selection

The study included patients who underwent cardiothoracic surgery between January 2009 to December 2016 in the Department of Cardiothoracic Surgery of The Centre for Cardiology & Cardiothoracic Surgery under the care of two surgeons. There were no changes in the surgeons practice except for the use of the ciNPT dressing for high risk patients. This is managed by the Hull University Teaching Hospitals NHS Trust which is a National Health Service (NHS) hospital that staffs over 600 inpatient beds. Patients who underwent cardiothoracic surgery in the 3 years (2009 to 2012) prior to the introduction of ciNPT (Control group, *n* = 927) and 3 years (2013 to 2016) after the introduction of ciNPT (ciNPT group, *n* = 932) were included. The high risk patients defined as patients with two or more of the previously identified risk factors constitue the study patients as they were the ones eligible to have the ciNPT dressing. There were 162 patients in the High Risk Control Group and 158 in the High Risk ciNPT Group. The latter were the only patients that had the ciNPT dressing applied post operatively. Eligible patients (> 18 years of age) were those undergoing an open-heart procedure that utilized a full median sternotomy (eg, coronary artery bypass grafting (CABG), CABG plus valve repair, valve repair solely, and other cardiac procedures). Patients who were at an elevated risk for developing SWI were included in the study [4]. All patients were followed p at 6 weeks following discharge and if they had no wound problems or on-going medical issues they were discharged to their General practitioner.

### Variables of interest

Patient risk factors included: obesity (body mass index [BMI] 103 > 32 kg/m2), age (> 80 years), chronic obtructive pulmonary disease (COPD), and diabetes mellitus. The logistic European System for Cardiac Operative Risk Evaluation (EuroSCORE) was calculated for patients within both cohorts to quantify the preoperative mortality risk profile. The two groups were compared using the following variables: age, obesity, COPD, diabetes, logistic EuroSCORE, and type of surgical procedure.

### Surgery

Cardiothoracic surgical procedures were performed via median sternotomy with either CABG, valve repair/replacement exclusively, CABG with valve repair/replacement, and other cardiac procedures. Classification of operative priority (Urgent, Emergency, Elective or Expedite) was also documented within data tables. All operations were performed with the use of cardiopulmonary bypass.

### Perioperative patient care and postoperative surgical wound management

All patients were administered antibiotics prophylactically according to the Trust antibiotic prophylaxis for surgical and invasive procedures guidelines for Cardiothoracic Surgery as shown in Table 1. Patients with suspected infections were started initially on Flucloxacillin (1 g intravenously); further treatment was guided by the results of microbiological assays that characterized the bacterial culture. The patients in the Control group received a transparent waterproof dressing with absorbent pad and bacterial barrier (OPSITE Post-Op; Smith & Nephew plc, Hull, UK) to their closed incisions. The dressing was left for 5 days providing the waterproof seal remained intact and the dressing was changed in cases of accumulation of hemoserous fluid or leak in the dressing. The patients in the study group received ciNPT (PREVENATM Incision Management System, KCI, an Acelity Company, San Antonio, TX, USA) applied to their sternal incision. The ciNPT dressing was changed after 5 days. In the event of a leak and loss of suction during the administration of ciNPT, another adhesive drape was added without exposing the surgical incision. All incisions were assessed after 5 days. Any sternal incisions demonstrating signs of infection were subject to bacterial swab cultures to identify the pathogen and antibiotic commenced. Post-sternotomy surgical incision infections were classified according to Jones et al. [6] that considered depth, anatomical site, and the degree of the infection (Table 2).

**Table 1:** Antibiotic prophylaxis for heart surgery – Coronary Artery Bypass Graft & Valve surgery as per Hospital Guidelines. GFR: Golerular Filtration Rate

Patients	Antibiotics
All Patients	Flucloxacillin 1 g IV at induction followed by 1 g IV after bypass (e.g. at skin closure) ***and then*** 6, 12 and 18 h later and then STOP ***plus*** Gentamicin 5 mg/kg IV (maximum 480 mg) at induction only **Note:** The administration of a 2nd (or sometimes 3rd) intra-operative dose of flucloxacillin will depend on factors such as the length of operation and the type of bypass used and is therefore at the discretion of the anaesthetist/surgeon to be adjusted according to circumstances
**Penicillin allergy**	Teicoplanin 400 mg IV (use 600 mg in those ≥90 kg) at induction with a subsequent dose at 12 h and then STOP ***plus*** Gentamicin 5 mg/kg IV (maximum 480 mg) at induction only
**Renal impairment**	Flucloxacillin 1 g IV: No adjustment required Gentamicin: 2 mg/kg if GFR < 50 ml/min Teicoplanin 400 mg IV: No adjustments required for single dose and if GFR > 60 ml/min. If GFR ≤60 ml/min – A 2nd dose within 24 h is NOT required

**Table 2:** Wound classification based on anatomical site plus a type including sepsis

CLASSIFICATION	DEPTH	DESCRIPTION
Type 1a	Superficial	Skin and subcutaneous
Type 1b	Superficial	Exposure of sutured deep fascia
Type 2a	Deep	Bone exposure, sternum with stable steel suture
Type 2b	Deep	Bone exposure, sternum with unstable steel suture
Type 3a	Deep	Necrotic bone exposure, or fractured, unstable sternum, exposed heart
Type 3b	Deep	Type 2 or 3 with septicemia

(Adapted from Jones et al. [6])

### Statistical methods/analysis

All data (patient demographics, EuroSCORE andLOS) were expressed as mean ± standard deviation and were compared between groups. The Fischer’s exact test of independence (for a two-tailed test) was used to calculate the *p*-value for all categorical data such as age, BMI, COPD, diabetes or number of infections. An unpaired, two-tailed t-test was used to calculate the *p*-value for all continuous data, such as the logistic EuroSCORE.

## Results

### Demographics data and risk factors

Table 3 compares the demographic and risk factors between the two groups. A total of 1859 patients were included in the study (Control group, *n* = 927; ciNPT group, *n* = 932).

**Table 3:** Patient Demographics, Perioperative Risk Factors, Operative Priority and Type of Cardiac Surgery

	**Overall Control Group (*n*** **= 927)**	**Overall ciNPT Group (*n*** **= 932)**	***P*** **value**
**RISK FACTORS**			
Age mean (SD)	67.8 (10.03)	67.1 (10.78)	0.1510
Male	680 (73.3%)	702 (75.3%)	
Female	247 (26.7%)	230 (24.7%)	
Obesity (BMI > 32 kg/m^2^)	207 (22.3%)	209 (22.4%)	1.00
COPD	166 (17.9%)	168 (18.0%)	1.000
Diabetes	236 (25.5%)	232 (24.9%)	0.836
**OTHER FACTORS**			
Logistic EuroSCORE mean ± SD	6.802 ± 9.7	8.3 ± 11.3	0.00015
**OPERATIVE PRIORITY**			
Urgent	217 (23.4%)	266 (28.5%)	0.0587
Emergency	14 (1.51%)	29 (3.11%)	0.0300
Elective	671 (72.4%)	610 (65.5%)	0.1678
Expedite	25 (2.7%)	28 (3.0%)	0.7810
**TYPE OF SURGERY**			
CABG	623 (67.2%)	607 (65.1%)	0.65
CABG + valve repair/replacement	65 (7.01%)	88 (9.44%)	0.09
Valve repair/replacement	130 (14.0%)	123 (13.2%)	0.68
Other cardiac procedure	109 (11.8%)	115 (12.3%)	0.77
	High Risk Control Group (*n* = 162)	High Risk ciNPT Group (*n* = 158)	
Age	70.4 ± 9.6	68.4 ± 9.9	0.06
Logistic EuroScore	9.1 ± 12.4	10.9 ± 12.8	0.17
Elective	127 (77%)	120 (76.5%)	
Urgent	33 (20%)	34 (21%)	
Expedite	4 (2.4%)	3 (1.9%)	
Emergency	1 (0.6%)	1 (0.6%)	

*SD* Standard Deviation, *BMI* Body Mass Index, *COPD* Chronic Obstructive Pulmonary Disease, *EuroSCORE* European System for Cardiac Operative Risk Evaluation, *CABG* Coronary Artery Bypass Grafting

Both groups had a similar average age, incidence of comorbidities (BMI, COPD, and diabetes), and type of surgery performed. These represent the overall group of patients but not the High Risk patients that are the study focus. For the Control and ciNPT cohorts the predicted operative mortality of patients was calculated using logistic EuroSCORE. The mean logistic EuroSCORE showed statistical significance between cohorts (Control group = 6.802 ± 9.7 vs. ciNPT group = 8.126 ± 11.3; *P* = 0.0002) There was no statistical difference in the operative priority as urgent, elective or expedite between the groups. Whereas, patients undergoingemergency surgery was higher (Control group = 14 vs. ciNPT group = 29; *P* = 0.0300) reflected statistical significance (Table 3). There was no statistical significance between types of surgeries (CABG, CABG + valve repair/replacement, valve repair/re placement only, or other) performed.

The patients either receiving or not the intervention were the patients with two or more risk factors and included 162 in the High Risk Control Group and 158 in the High Risk ciNPT Group. There were no differences identified between these two groups in any of the cahracteristics compared including logistic Euroscore.

### Postoperative distribution of SWI between cohorts

The distribution of SWI among the overall patients is shown in Table 4. Of the 927 parents in the Control group, a total of 81 (8.7%) patients had a SWI. Of these, 63 patients had a SWI classified as a superficial infection; 17 patients required debridement and 8 patients had poststernotomy incisions that required resuturing. Of the 932 patients in the ciNPT group, a total of 41 (4.4%) patients had a SWI. Thirty-six patients had a SWI classified as a superficial infection; 5 patients required debridement and 3 patients required sternal incision resuturing. The incidence of SWI between the Control and ciNPT groups, 81 (8.7%) patients vs. 41 (4.4%), respectively was statistically significant (*P* = 0.0005). However the incidence.

**Table 4:** Distribution of Sternal Wound Infections (SWI) among patients receiving conventional dressings or ciNPT

	Incidence of SWI among cohort participants				
	Overall Control Group (*n* = 927)		Overall ciNPT Group (*n* = 932)		*P* value
Total Patients with SWI	81 (8.7%)		41 (4.4%)		0.0005
Superficial SWI	63 (6.8%)		36 (3.9%)		0.0001
SWI requiring debridement	17 (1.8%)		5 (0.54%)		0.0001
SWI requiring sternal resuturing	8 (0.86%)		3 (0.32%)		0.004
**Postoperative LOS (days; mean ± SD)**					
	Control Group		ciNPT Group		
No Infection	9.04 ± 5.78		11.4 ± 5.43		0.0001
Superficial SWI	19.3 ± 3.65		21.2 ± 16.49		0.009
SWI requiring debridement	66.7 ± 10.53		54.0 ± 35.03		0.01
SWI requiring sternal resuturing	55.0 ± 17.75		53.8 ± 19.6		0.22
**Inhospital mortality Overall Groups**					
	Control Group		ciNPT Group		
	Deceased	Alive	Deceased	Alive	
Superficial SWI	0	63	0	36	
SWI requiring debridement	1	16	0	5	
SWI requiring sternal resuturing	1	7	0	3	
	High Risk Control Group *n* = 162		High Risk ciNPT Group *n* = 158		
SWI	20 (12.3%)		9 (5.6%)		0.049
Length of post stay	13.0 ± 15.1		12.2 ± 15.6		0.65

Standard Deviation (SD); Sternal Wound Infection (SWI), Length of Stay (LOS)

### Infection rates among patients with ≥2 risk factors

**Fig. 1:**
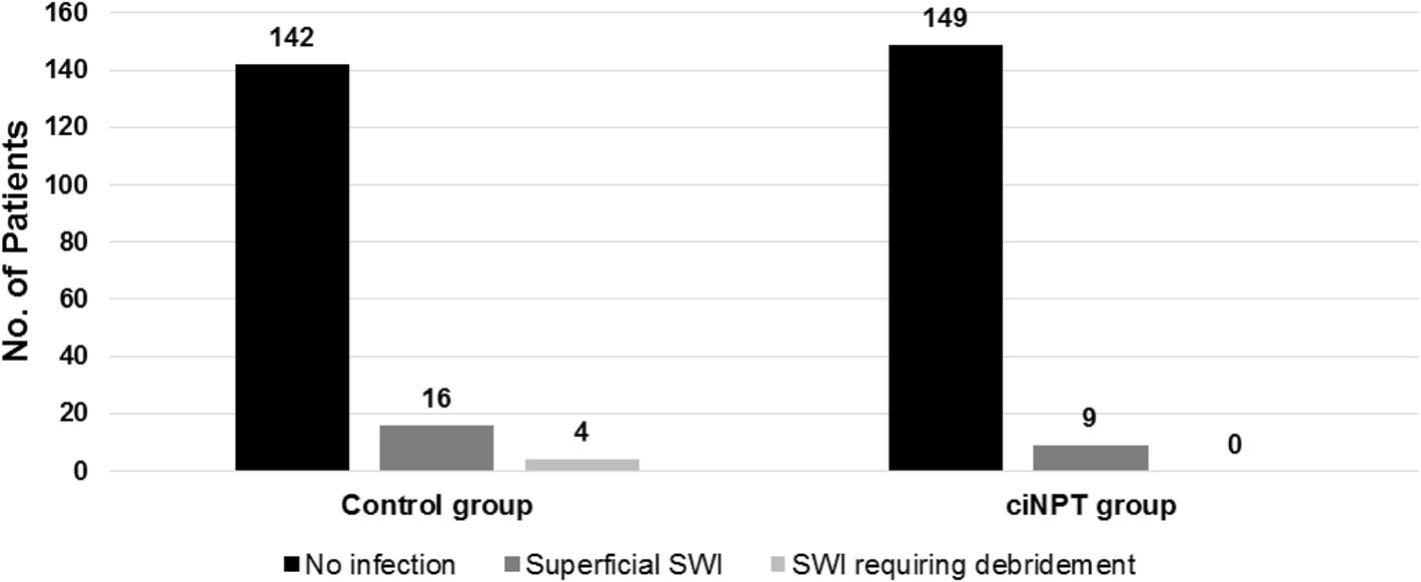
Sternal Site Infection Rates Among Patients with ≥2 Risk Factors. Patients with ≥2 Risk Factors who received ciNPT had a lower incidence of developing a sternal wound infection and did not require debridement

In the High Risk Control group, 162 (17.5%) had 2 or more risk factors. Amongthese patients, 16 presented with superficial SWI, while 4 required debridement (Fig. 1). Conversely, 158 patients (17%) within the ciNPT group (High Risk ciNPT) patients exhibited 2 or more risk factors; however, 9 of these patients only developed a superficial SWI and none required debridement which was statistically significant (*P* = 0.049).

### Length of inpatient hospital stay

**Fig. 2:**
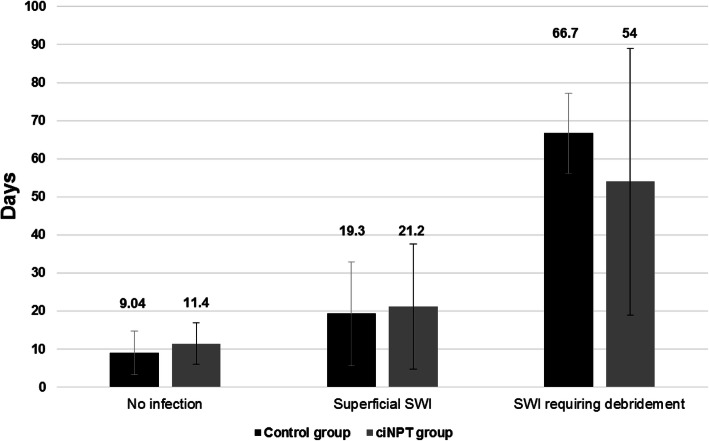
Mean Length of Inpatient Hospital Stay

The ciNPT group demonstrated a higher mortality risk profile evidenced by a higher mean logistic EuroSCORE, and further reflected by the higher number of patients who had emergency surgery. Table 4 also captures the mean length of postoperative inpatient stay (LOS) for patients receiving in th e Overall Groups ofconventional dressings or ciNPT. Patients with no infection had a longer mean hospital stay (11.4 ± 5.43 vs 9.04 ± 5.78 days; *P* = 0.0001) in the ciNPT group compared with the control group (Fig. 2).

Similarly, patients with superficial SWI in the ciNPT group had a longer mean hospital stay compared with the control group (21.2 ± 16.49 days vs 19.3 ± 13.65 days; *P* = 0.0096; Fig. 2). However, patients in the control group with SWI requiring debridement had a statistically significant longer hospital stay (66.7 ± 10.53 vs 54.0 ± 35.03 days; *P* = 0.0102) when compared to the study patients (Fig. 2). On the other hand, there was no difference between the two groups for patients with SWI requiring sternal resuturing (55.0 ± 17.75 vs 53.8 ± 19.6; *P* = 0.2252). The High Risk Patients had similar postoperative length (13.0 ± 15.1 versus 12.2 ± 15.6 days; *P* = 0.65). However the patients that developed infections in the two High Risk Groups stayed significantly longer than those who did not (25.5 ± 27.7 versus 12.2 ± 15.6 days;*P* = 0.008). There were 13 deaths in Hospital in the High Risk Control Group versus 10 in the High Risk ciNPT Group (*P* = 0.66).

## Discussion

The clinical and economic burden of wound infection particularly in cardiac surgery has shifted focus to prevention rather than treatment alone [7]. This clinical investigation was a retrospective study to evaluate the clinical benefit of ciNPT among high risk patients who underwent cardiac surgery. In our dataset, we have demonstrated that the application of ciNPT to manage sternal incisions among patients with ≥2 risk factors may lower the incidence of SWIs with patients having longer postoperative stay if they develop an infection..

The absence of consensus concerning the clinical efficacy and broader adoption of ciNPT after median sternotomy is constrained by several factors, such as the dearth of medical literature and the lack of large and robust health economic studies. Incision management modalities that employ subatmospheric pressure are generally more expensive than standard care, and controversy arises as to whether the wider use of ciNPT would significantly mitigate SSI risk and reduce the economic burden posed by post-sternotomy SSIs.

SWI is a debilitating postoperative complication for patients, and we have reported previously an incidence of DSWI of 0.59% with an associated 1-year mortality rate of 9.1% with older age, obesity, diabetes or respiratory compromise as risk factors [4]. According to consensus recommendations by Willy et al. [8], obesity (BMI ≥ 30 kg/m2), diabetes mellitus, respiratory insufficiency, and tobacco use were among the most common patient comorbid risk factors that elevate the risk of SSI development. In their two-centre (*n* = 996 patients) randomized controlled trial (RCT), Schimmer et al. [9] identified a preoperative BMI > 30 kg/m2 as an independent predictor for an increased rate of sternal surgical site infections (SSI). However, Allen et al. [10] demonstrated via a multivariate exact logistic regression model that BMI was not a significant predictor of sternal complications and SSIs. Our previous work identified 4 risk factors (age, BMI, COPD and diabetes) that elevated the incidence of post-sternotomy SWIs [4]. The patient is considered high-risk for developing SWI if they have 2 out of the 4 risk factors identified [4]. Diabetes and obesity represented the most common comorbidities in the study cohorts; however, patient demographics and comorbidities were similar between control and ciNPT cohorts. The current study also takes into consideration a patient’s critical preoperative state by calculating the logistic EuroSCORE to assign a preoperative mortality risk grade. Although we had identified a statistical significance comparing mean logistic EuroSCORE of the overall control cohort versus the ciNPT study cohorts this was not the case for the High Risk Groups.

Select surgical procedures present an elevated risk of delayed healing that can foster the development of an SSI. As sternotomy is characterized as a high-risk incision, complications after CABG and the harvest of bilateral internal thoracic arteries or mammary arteries can be considered operation-related risk factors [9, 11]. CABG was the most common cardiac procedure in our study indicating an elevated surgical risk factor of the development of SWI in our study population.

Few studies have been conducted on the use of ciNPT as a preventative measure in cardiac surgery, although they have yielded positive results. Some of these studies focused on the high-risk population group [12, 13]. Agarwal and colleagues [14] recommended that ciNPT be considered as a standard treatment for patients with SWI.

However, this trial focussed solely on treating patients, who had developed complication with the sternal incision, as opposed to mitigating the incidence of this complication [14].

Grauhan et al. [5] assessed the effect of ciNPT on incision management in 150 consecutive obese patients undergoing sternotomy. The patients were split into two groups, those who received conventional dressing (*n* = 75) and those who received ciNPT (*n* = 75) [5]. Significantly lower rates of infection were reported in the ciNPT group compared to the control group [5]. Grauhan et al. [15] followed up this research with a further trial looking at the effect of ciNPT in a general population of post-sternotomy patients (*n* = 237) compared to a historical cohort that received conventional dressings (*n* = 3508). The authors found the ciNPT group had a significantly lower infection rate compared to the historical control group [15]. The authors found that the ciNPT group had a significantly lower infection rate compared to the historical group (1.3% for ciNPT vs. 3.4% for control; *P* ≤ 0.05) [15]. In our study, ciNPT had a lower infection rate among patients with 2 or more risk factors Our results appear to match those reported in the current published literature. The positive impact of the ciNPT might be a result of enhanced tissue perfusion facilitated by applying ciNPT to the sternal incision which may promote healing and sequestere the incision from external contamination; thereby, accounting for the reduced incidence of SWI among ciNPT patients.

The management of deep SWI after cardiac surgery represents a persistent challenge and can extend hospitalization. Protracted length of hospitalization is associated with the incidence of SSIs, evidenced by patients affected with an SSI after undergoing cardiac surgery spending an additional 23 days in hospital [16]. In a review of data of 999 consecutive patients who underwent coronary artery bypass grafting (CABG) during a 24-month period, Findeisen et al. [17] evaluated protracted LOS due to SSIs.

The SSI-related mean additional LOS was estimated to be 9.3 days [17]. In a retrospective, nonrandomized review of patients receiving median sternotomy procedures, Miyahara et al. [18] reported significantly longer length of intensive care unit (ICU) stay and hospital LOS in patients with deep SWI. In our study, the mean extended LOS for ciNPT was shorter than the control group (11.4 days for ciNPT vs. 218.4 days for control). Further, we report that the mean post-operative LOS of patients with superficial SWI in the ciNPT group was longer, which might be accounted for by the larger number of acute patients enrolled in the ciNPT group as evidenced by an elevated EuroSCORE. There was no further analysis done to identify whether the patients experienced a longer LOS due to ciNPT use or patient EuroSCORE.

Poststernotomy surgical site occurrences, particularly SSIs exert a significant pecuniary impact on individual patients, healthcare systems, and resource utilization. Not all patients require ciNPT, individuals at high-risk for complications have been reported to receive cost-effective clinical benefit from ciNPT use [10, 17]. Nussbaum et al. [19] characterized surgical infection and surgical wounds among the most prevalent and the most expensive (ranging from $11.7 to $38.3 billion) wound aetiologies. Individual Medicare spending per wound costing $3364 + $2604 ($5968) to $14,153 + $6585 ($20,738) for Medicare beneficiaries in 2014 [19]. Limited evidence exists regarding economics aspects of ciNPT use in the post-sternotomy population. Grauhan et al. [15] considered the economic aspects of ciNPT in post-sternotomy patients. Their report suggested that comprehensive use of ciNPT in Germany would yield an annual cost of approximately €30,000,000, which would represent an appreciable reduction from the avoidable economic loss of €60,000,000 to €90,000,000 associated with patients with 302 wound infections requiring surgical revision [15]. Citing the additional 23 days in hospital and additional cost of £11,003.31 for SSI following cardiac surgery, Philip et al. [16] recommended ciNPT be given consideration for patients at risk of developing post-sternotomy SSIs. This was compared to three representative cases of post-sternotomy SSIs at their institution where patient LOS was extended by 12–25 days and additional costs ranging from €12,214 to €22,456 [16]. This is obviously can be extended to our patient population although we have not conducted a detailed financial implication analysis.

### Limitations

The major limitation of this study is that it compares consecutive rather than contemporary patient cohorts. This introduces the potential of time-effect bias, related to unmeasured changes in the care of patients over the course of the years of the study. However it has to be stated that there were no changes in the surgical protocols or antibiotic regimen in the period studied and the surgeons involved introduced no other changes to their practice. That said it should be noted that there was an overall reduction in the incidence of SWI between the two overall groups although only the high risk patients received the ciNPT.

Another possible limitation is that consideration was not afforded to microbiological characterization of the infected wound and the sensitivity of infecting agents.. Furthermore we had not conducted a comprehensive cost analysis of the impact of infection and wherther the use of the ciNPT mitigates that. Future studies using improved models can be designed for sensitive capture of quality-adjusted life years (QALY) and studies incorporating sensitivity analysis may assist in better defining cost offsets and health economic endpoints to mitigate the uncertainty of the cost-effectiveness and clinical efficacy of ciNPT in managing post sternotomy incisions.

## Conclusion

SWI following cardiac surgery is a recognised major complication. In this study, ciNPT reduced the incidence of SWI after median sternotomy in patients at an elevated risk for developing SWI. Reduced SWI incidence may result in a shorter inpatient hospital stay, a decreased rate of mortality, and tentatively reduced financial cost although not demonstrated in this study. Nonetheless, our results indicate that ciNPT can be a valuable tool in reducing the incidence of SWI in high-risk patients.

## Data Availability

All data generated or analysed during this study are included in this published article and are available from the corresponding author on reasonable request.

## Abbreviations

BMI: Body mass index
CABG: Coronary artery bypass grafting
ciNPT: Closed incision negative pressure therapy
COPD: Chronic obstructive pulmonary disease
EuroSCORE: European System for Cardiac Operative Risk Evaluation
GFR: Glomerular filtration rate
ICU: Intensive care unit
IV: Intravenous
LOS: Length of stay
NHS: National Health Service
QALY: Quality-adjusted life year
RCT: Randomized controlled trial
SD: Standard deviation
SSI: Surgical site infections
SWI: Sternal wound infection
